# Supplementary material for: Community-directed treatment with ivermectin in Maridi, South Sudan: Impact of an onchocerciasis awareness campaign and bi-annual treatment on therapeutic coverage
Source: PLoS Negl Trop Dis. 2025 Sep 8;19(9):e0013493. doi: 10.1371/journal.pntd.0013493 (PMC12431650; doi:10.1371/journal.pntd.0013493)
Supplement: S1 File — (PDF) [file pntd.0013493.s001.pdf]

HOUSEHOLD SURVEY QUESTIONNAIRE: MORTALITY AND IVERMECTIN

Investigator's Name: ______________________________ Signature:

Village: ______________________ Date (DD/MM/YYYY): __ __/__ __/__ __ __ __

Household Characteristics:

HOUSEHOLD CODE (I/VV/HHH): __ __ / __ __ /__ __ __ (I = Investigator code; VV = Village code; HHH = Household number)

1) Name of Household head **(*)**: ______________________________ Tel: ______________________

2) Does the family originate from this village?: ☐ YES ☐ NO If NO, How long have they been residing in this village? __________ (years)

3) Main income generating activity for the family:
☐ Farming ☐ Cattle rearer ☐ Fishing ☐ Employee ☐ Craftsman ☐ Other, specify: ______________________

4) During the past two years (2022 and 2023), did someone die in this household? ☐ YES ☐ NO ☐ DON'T KNOW

If YES, Year: ☐ 2022 ☐ 2023 Sex (M/F): _____ Age at death: ____years Epilepsy: ☐ Yes ☐ No Cause of death: ____________________
If YES, Year: ☐ 2022 ☐ 2023 Sex (M/F): _____ Age at death: ____years Epilepsy: ☐ Yes ☐ No Cause of death: ____________________
If YES, Year: ☐ 2022 ☐ 2023 Sex (M/F): _____ Age at death: ____years Epilepsy: ☐ Yes ☐ No Cause of death: ____________________
If YES, Year: ☐ 2022 ☐ 2023 Sex (M/F): _____ Age at death: ____years Epilepsy: ☐ Yes ☐ No Cause of death: ____________________
If YES, Year: ☐ 2022 ☐ 2023 Sex (M/F): _____ Age at death: ____years Epilepsy: ☐ Yes ☐ No Cause of death: ____________________

(*) Household definition: *All occupants of the same home with or without parental ties who take meals together*

EPILEPSY SCREENING:

1. No. pers.		2. Age	3. Gender		Epilepsy/Nodding Syndrome (Followed-up at Maridi Hospital)		Took IVM in MAY 2023		IF NO IVM in May 2023, Reason?	Took IVM in NOVEMBER 2023		IF NO IVM in NOVEMBER 2023, Reason?	NEVER TOOK IVM	ITCHING	VISUAL ACUITY
Pst	Abs	In years (Y) or Months (M)	M=Male F=Female		Yes	No	Yes	No	Write the reason	Yes	No	Write the reason	Mark X	Y / N	Good / Poor/ Blind
1 ☐	☐		M ☐	F ☐	☐	☐	☐	☐		☐	☐				
2 ☐	☐		M ☐	F ☐	☐	☐	☐	☐		☐	☐				
3 ☐	☐		M ☐	F ☐	☐	☐	☐	☐		☐	☐				
4 ☐	☐		M ☐	F ☐	☐	☐	☐	☐		☐	☐				
5 ☐	☐		M ☐	F ☐	☐	☐	☐	☐		☐	☐				
6 ☐	☐		M ☐	F ☐	☐	☐	☐	☐		☐	☐				
7 ☐	☐		M ☐	F ☐	☐	☐	☐	☐		☐	☐				
8 ☐	☐		M ☐	F ☐	☐	☐	☐	☐		☐	☐				
9 ☐	☐		M ☐	F ☐	☐	☐	☐	☐		☐	☐				
10 ☐	☐		M ☐	F ☐	☐	☐	☐	☐		☐	☐				
11 ☐	☐		M ☐	F ☐	☐	☐	☐	☐		☐	☐				
12 ☐	☐		M ☐	F ☐	☐	☐	☐	☐		☐	☐				
13 ☐	☐		M ☐	F ☐	☐	☐	☐	☐		☐	☐				
14 ☐	☐		M ☐	F ☐	☐	☐	☐	☐		☐	☐				
15 ☐	☐		M ☐	F ☐	☐	☐	☐	☐		☐	☐				
16 ☐	☐		M ☐	F ☐	☐	☐	☐	☐		☐	☐				
17 ☐	☐		M ☐	F ☐	☐	☐	☐	☐		☐	☐				
18 ☐	☐		M ☐	F ☐	☐	☐	☐	☐		☐	☐				
19 ☐	☐		M ☐	F ☐	☐	☐	☐	☐		☐	☐				
20 ☐	☐		M ☐	F ☐	☐	☐	☐	☐		☐	☐				

PERSONS AGED 10 YEARS AND ABOVE WHO NEVER TOOK IVERMECTIN

CODE (I/VV/HHH/N°): __ __/__ __/__ __ __/__ __ Reason: ____________________

CODE (I/VV/HHH/N°): __ __/__ __/__ __ __/__ __ Reason: ____________________

CODE (I/VV/HHH/N°): __ __/__ __/__ __ __/__ __ Reason: ____________________

CODE (I/VV/HHH/N°): __ __/__ __/__ __ __/__ __ Reason: ____________________

CODE (I/VV/HHH/N°): __ __/__ __/__ __ __/__ __ Reason: ____________________
