# Supplementary material for: Community-directed treatment with ivermectin in Maridi, South Sudan: Impact of an onchocerciasis awareness campaign and bi-annual treatment on therapeutic coverage
Source: PLoS Negl Trop Dis. 2025 Sep 8;19(9):e0013493. doi: 10.1371/journal.pntd.0013493 (PMC12431650; doi:10.1371/journal.pntd.0013493)
Supplement: S2 File — (DOCX) [file pntd.0013493.s002.docx]

**Supplementary File 2.** Reasons for not taking ivermectin during the previous CDTI round

| **Reason for not receiving drug** | **Frequency (n=326)** | **%** |
| --- | --- | --- |
| I was too far away | 78 | 23.9 |
| I was pregnant | 48 | 14.7 |
| I was under-age | 35 | 10.7 |
| The CDD did not come to my house/school/fixed point location | 34 | 10.4 |
| I was afraid of side-effects | 23 | 7.1 |
| I was ill | 22 | 6.7 |
| I was busy / not available | 18 | 5.5 |
| I was taking other medications | 14 | 4.3 |
| I was breastfeeding | 12 | 3.7 |
| I was unaware that CDTI was going on | 11 | 3.4 |
| The drugs were finished when I wanted to take them | 7 | 2.1 |
| I am not at risk for this disease | 6 | 1.8 |
| The drug does not work | 1 | 0.3 |
| I am too old | 1 | 0.3 |
| Other reasons | 16 | 4.9 |
| **Total** | **326** | **100** |
