# Supplementary material for: Community-directed treatment with ivermectin in Maridi, South Sudan: Impact of an onchocerciasis awareness campaign and bi-annual treatment on therapeutic coverage
Source: PLoS Negl Trop Dis. 2025 Sep 8;19(9):e0013493. doi: 10.1371/journal.pntd.0013493 (PMC12431650; doi:10.1371/journal.pntd.0013493)
Supplement: Attachment — Submitted filename: 16.07.25Response to reviewers PNTD Maridi IVMRC+JS (002).docx [file pntd.0013493.s005.docx]

**Email from PLOS NTD**

Please include the following items when submitting your revised manuscript:

* A rebuttal letter that responds to each point raised by the editor and reviewer(s). You should upload this letter as a separate file labeled 'Response to Reviewers'. This file does not need to include responses to any formatting updates and technical items listed in the 'Journal Requirements' section below.

* A marked-up copy of your manuscript that highlights changes made to the original version. You should upload this as a separate file labeled 'Revised Manuscript with Track Changes'.

* An unmarked version of your revised paper without tracked changes. You should upload this as a separate file labeled 'Manuscript'.

If you would like to make changes to your financial disclosure, competing interests statement, or data availability statement, please make these updates within the submission form at the time of resubmission. Guidelines for resubmitting your figure files are available below the reviewer comments at the end of this letter.

**RESPONSE TO REVIEWERS**

**Journal Requirements:**

1) Please ensure that the CRediT author contributions listed for every co-author are completed accurately and in full.
At this stage, the following Authors/Authors require contributions: Robert Colebunders. Please ensure that the full contributions of each author are acknowledged in the "Add/Edit/Remove Authors" section of our submission form.
The list of CRediT author contributions may be found here: [https://journals.plos.org/plosntds/s/authorship#loc-author-contributions](https://eur01.safelinks.protection.outlook.com/?url=https%3A%2F%2Fjournals.plos.org%2Fplosntds%2Fs%2Fauthorship%23loc-author-contributions&data=05%7C02%7CJosephNelson.SieweFodjo%40uantwerpen.be%7C416708a876e6421c666608ddbeedd579%7C792e08fb2d544a8eaf72202548136ef6%7C0%7C0%7C638876650885150809%7CUnknown%7CTWFpbGZsb3d8eyJFbXB0eU1hcGkiOnRydWUsIlYiOiIwLjAuMDAwMCIsIlAiOiJXaW4zMiIsIkFOIjoiTWFpbCIsIldUIjoyfQ%3D%3D%7C0%7C%7C%7C&sdata=tOkBzLSACHACdWZgg6RLpSEtQckaoInOJwAqWkZE%2Fl0%3D&reserved=0)

**Response: Done**

2) Please upload all main figures as separate Figure files in .tif or .eps format. For more information about how to convert and format your figure files please see our guidelines: 
[https://journals.plos.org/plosntds/s/figures](https://eur01.safelinks.protection.outlook.com/?url=https%3A%2F%2Fjournals.plos.org%2Fplosntds%2Fs%2Ffigures&data=05%7C02%7CJosephNelson.SieweFodjo%40uantwerpen.be%7C416708a876e6421c666608ddbeedd579%7C792e08fb2d544a8eaf72202548136ef6%7C0%7C0%7C638876650885166893%7CUnknown%7CTWFpbGZsb3d8eyJFbXB0eU1hcGkiOnRydWUsIlYiOiIwLjAuMDAwMCIsIlAiOiJXaW4zMiIsIkFOIjoiTWFpbCIsIldUIjoyfQ%3D%3D%7C0%7C%7C%7C&sdata=2tY3NzVTu5fB%2FBm%2B4JX9GWJBZMPoop%2FY7%2BTz2OBNcU0%3D&reserved=0)

**Response: Done**

3) Some material included in your submission may be copyrighted. According to PLOSu2019s copyright policy, authors who use figures or other material (e.g., graphics, clipart, maps) from another author or copyright holder must demonstrate or obtain permission to publish this material under the Creative Commons Attribution 4.0 International (CC BY 4.0) License used by PLOS journals. Please closely review the details of PLOSu2019s copyright requirements here: PLOS Licenses and Copyright. If you need to request permissions from a copyright holder, you may use PLOS's Copyright Content Permission form.
Please respond directly to this email and provide any known details concerning your material's license terms and permissions required for reuse, even if you have not yet obtained copyright permissions or are unsure of your material's copyright compatibility. Once you have responded and addressed all other outstanding technical requirements, you may resubmit your manuscript within Editorial Manager. 
Potential Copyright Issues:

- Please confirm (a) that you are the photographer of Supplementary File 1, or (b) provide written permission from the photographer to publish the photo(s) under our CC BY 4.0 license.

**Response: Photos in** **Supplementary File 1 were taken by co-authors on this publication who have given their permission. The photo credits have now been added in the Supplementary material.**

- Figures 1 and 3. Please (a) provide a direct link to the base layer of the map (i.e., the country or region border shape) and ensure this is also included in the figure legend; and (b) provide a link to the terms of use / license information for the base layer image or shapefile. We cannot publish proprietary or copyrighted maps (e.g. Google Maps, Mapquest) and the terms of use for your map base layer must be compatible with our CC BY 4.0 license.
Note: if you created the map in a software program like R or ArcGIS, please locate and indicate the source of the basemap shapefile onto which data has been plotted.
If your map was obtained from a copyrighted source please amend the figure so that the base map used is from an openly available source. Alternatively, please provide explicit written permission from the copyright holder granting you the right to publish the material under our CC BY 4.0 license.
If you are unsure whether you can use a map or not, please do reach out and we will be able to help you. The following websites are good examples of where you can source open access or public domain maps:
* U.S. Geological Survey (USGS) - All maps are in the public domain. ([http://www.usgs.gov](https://eur01.safelinks.protection.outlook.com/?url=http%3A%2F%2Fwww.usgs.gov%2F&data=05%7C02%7CJosephNelson.SieweFodjo%40uantwerpen.be%7C416708a876e6421c666608ddbeedd579%7C792e08fb2d544a8eaf72202548136ef6%7C0%7C0%7C638876650885192495%7CUnknown%7CTWFpbGZsb3d8eyJFbXB0eU1hcGkiOnRydWUsIlYiOiIwLjAuMDAwMCIsIlAiOiJXaW4zMiIsIkFOIjoiTWFpbCIsIldUIjoyfQ%3D%3D%7C0%7C%7C%7C&sdata=1uCxO6HAbEVr4gulKyMsagyngAzaXj5uydvlS1LodLM%3D&reserved=0))
* PlaniGlobe - All maps are published under a Creative Commons license so please cite u201cPlaniGlobe, [http://www.planiglobe.com](https://eur01.safelinks.protection.outlook.com/?url=http%3A%2F%2Fwww.planiglobe.com%2F&data=05%7C02%7CJosephNelson.SieweFodjo%40uantwerpen.be%7C416708a876e6421c666608ddbeedd579%7C792e08fb2d544a8eaf72202548136ef6%7C0%7C0%7C638876650885218369%7CUnknown%7CTWFpbGZsb3d8eyJFbXB0eU1hcGkiOnRydWUsIlYiOiIwLjAuMDAwMCIsIlAiOiJXaW4zMiIsIkFOIjoiTWFpbCIsIldUIjoyfQ%3D%3D%7C0%7C%7C%7C&sdata=JV7mssxC6YSoQosozk19YHtZRvbxLUU%2Fne19dsU21UE%3D&reserved=0), CC BY 2.0u201d in the image credit after the caption. ([http://www.planiglobe.com/?lang=enl](https://eur01.safelinks.protection.outlook.com/?url=http%3A%2F%2Fwww.planiglobe.com%2F%3Flang%3Denl&data=05%7C02%7CJosephNelson.SieweFodjo%40uantwerpen.be%7C416708a876e6421c666608ddbeedd579%7C792e08fb2d544a8eaf72202548136ef6%7C0%7C0%7C638876650885244765%7CUnknown%7CTWFpbGZsb3d8eyJFbXB0eU1hcGkiOnRydWUsIlYiOiIwLjAuMDAwMCIsIlAiOiJXaW4zMiIsIkFOIjoiTWFpbCIsIldUIjoyfQ%3D%3D%7C0%7C%7C%7C&sdata=HEgB%2FuTDphvyljyfbvA1bjCIrEMoZEVpfJJfYTb4Rf4%3D&reserved=0))
* Natural Earth - All maps are public domain. ([http://www.naturalearthdata.com/about/terms-of-use/](https://eur01.safelinks.protection.outlook.com/?url=http%3A%2F%2Fwww.naturalearthdata.com%2Fabout%2Fterms-of-use%2F&data=05%7C02%7CJosephNelson.SieweFodjo%40uantwerpen.be%7C416708a876e6421c666608ddbeedd579%7C792e08fb2d544a8eaf72202548136ef6%7C0%7C0%7C638876650885271566%7CUnknown%7CTWFpbGZsb3d8eyJFbXB0eU1hcGkiOnRydWUsIlYiOiIwLjAuMDAwMCIsIlAiOiJXaW4zMiIsIkFOIjoiTWFpbCIsIldUIjoyfQ%3D%3D%7C0%7C%7C%7C&sdata=3Rupjno5%2B%2B%2FPWyikT9M5ZIspaVxZt29l8K82e5nCSlk%3D&reserved=0)).

**Response: Figure 1 was taken from Wikimedia and is free for use, both for commercial and non-commercial purposes, provided the source is acknowledged (Reference [24] in Manuscript, also provides the link to the image source). Figure 3 was drawn by the Maridi County offices (not from basemaps incorporated in a given software). Figure 3 was previously published in another research article and must not be included in the current manuscript. We there removed it and now direct the readers to the published article containing this same map. Therefore there are no copyright issues.**

4) In the online submission form, you indicated that "Data are available with the authors by a simple request". All PLOS journals now require all data underlying the findings described in their manuscript to be freely available to other researchers, either
- In a public repository
- Within the manuscript itself
- Uploaded as supplementary information.
This policy applies to all data except where public deposition would breach compliance with the protocol approved by your research ethics board. If your data cannot be made publicly available for ethical or legal reasons (e.g., public availability would compromise patient privacy), please explain your reasons by return email and your exemption request will be escalated to the editor for approval. Your exemption request will be handled independently and will not hold up the peer review process, but will need to be resolved should your manuscript be accepted for publication. One of the Editorial team will then be in touch if there are any issues.

**Response: We will upload the data as supplementary file to this article. See attached Excel sheet (Supplementary File 4).**

5) Please ensure that the funders and grant numbers match between the Financial Disclosure field and the Funding Information tab in your submission form. Note that the funders must be provided in the same order in both places as well. State the initials, alongside each funding source, of each author to receive each grant. For example: "This work was supported by the National Institutes of Health (####### to AM; ###### to CJ) and the National Science Foundation (###### to AM).".
If you did not receive any funding for this study, please simply state: u201cThe authors received no specific funding for this work.u201d

**Response: The funding information is correct**

**REVIEWERS' COMMENTS:**

**Reviewer #1**:

Overall the methods are clearly stated and appropriate.
It is not mentioned if the survey approaches/field work needed to be adapted due to insecurity. If they did then this should be documented as an important contribution to these types of areas - which should also flow to results and methods.

**Response: Insecurity was mentioned in the introduction as a factor complicating the implementation of CDTI. However, no significant insecurity was noted in the Maridi area during the study period, therefore we did not deem it necessary to adapt our fieldwork approaches in that respect. Nevertheless, our paper provides practical approaches for IVM distribution that are applicable in other resource-limited (humanitarian) settings across Africa.**

The results follow the methods well & clearly presented.
Figure 3 seems to be obtained from another source - can this be clarified.

**Response: Figure 3 has now been removed.**

Overall the conclusions reflect the findings and present a range of important issues re social mobilisation, reporting data/coverage surveys/ epilepsy. What is missing is how they did this or needed to adapt the strategies in a conflict/insecure environment. There is little information on this to fully appreciate the context.

**Response: While the research occurred in a post-conflict setting, no insecurity was noted during the study period. The IVM distribution / survey approaches described in our study are mostly community-based because this is the best strategy to not to leave anyone behind in poor and humanitarian settings.**

Overall a well written and important paper on factor influencing the increase in MDA for onchocerciasis in South Sudan. It covers many important topics. More on the conflict/insecure environment and how it may have impacted any of the activities would be useful to include as this is a neglected area of NTD research and the more information that is gathered and published, the more that practical strategies may be developed from lessons learned. I don't envisage this addition to be extensive but needs more present to appreciate the context.

**Response: In the introduction, we mention that “insecurity was hampering the yearly implementation of CDTI but also limited funding, as well as inadequate numbers and insufficient supportive supervision of community drug distributors (CDDs) [17,18].” In the revised version of the paper we now added “In 2006, around 40% (4.1 million) of the South Sudan’s population were at risk of onchocerciasis, of which 3.6 million were eligible for CDTI However, only 26% of the eligible population received treatment with ivermectin in the five CDTI areas”**

**Reviewer #2**:

Fodjo et al. evaluated the impact of onchocerciasis awareness programs combined with biannual ivermectin treatment (CDTI) in the Maridi region of South Sudan. The study demonstrated that integrating reinforced community awareness with biannual CDTI significantly increased ivermectin treatment coverage. This combined approach proved more effective than standard strategies and presents a promising avenue for advancing onchocerciasis elimination efforts in remote and conflict-affected areas of South Sudan.

Major Concerns:

1) Several studies, including some from the same research group, have already reported on the outcomes of awareness programs and biannual CDTI in other countries. However, these publications are not cited, and no comparison is made between findings across different settings. A broader contextualization would strengthen the manuscript and highlight the relevance of the results beyond South Sudan.
**Response:** **We thank the reviewer for this comment, and we agree to broaden this discussion by mentioning other settings where CDTI was improved and the resulting benefits that were achieved. We now added in the discussion section “[43]. In Maridi, the switch from annual to bi-annual ivermectin distribution with improved therapeutic coverage resulted in >80% decrease in the incidence of OAE including nodding syndrome [25], much higher than the 50% decrease predicted during the protocol phase [20]. Similar drops in OAE incidence were also observed in other onchocerciasis-endemic settings like in the Mvolo County of South Sudan after introducing annual CDTI [28], and in the Mahenge area of Tanzania after switching to bi-annual CDTI [44].”**

2) The same research group previously published a study protocol for biannual CDTI in the same region, but this earlier work is neither cited nor discussed. The manuscript should clarify the relationship between the current study and the published protocol.

**Response: This protocol is now mentioned in the discussion (reference [20]), and we highlight that the observed findings even exceeded the expectations that were penned down in the protocol paper: “In Maridi, the switch from annual to bi-annual ivermectin distribution with improved therapeutic coverage resulted in >80% decrease in the incidence of OAE including nodding syndrome [25], much higher than the 50% decrease predicted during the protocol phase [20].”**

**In our manuscript, we also make reference to the paper by Jada et al (**[**https://doi.org/10.1186/s40249-018-0496-2**](https://doi.org/10.1186/s40249-018-0496-2)**) as reference [25]. This reference [25] documents the changes in epilepsy incidence following biannual CDTI and other onchocerciasis elimination efforts in Maridi. Both the current manuscript and the paper by Jada et al. present complementary findings from the published protocol.**

3) Figure 3 is blurry and difficult to interpret. A higher-resolution version should be provided to ensure clarity and readability.

**Response: Figure 3 has been removed from the manuscript.**

4) The full questionnaires used in the study should be included in the supplementary materials to allow for transparency and reproducibility.

**Response: Questionnaires added as Supplementary File 3.**

5) The authors note that compliance with ivermectin treatment is higher among individuals affected by nodding syndrome (NS) or epilepsy. It would be important to also address other clinical manifestations of onchocerciasis, such as blindness or hyperreactive onchodermatitis. How many individuals in the study area suffer from these or other onchocerciasis-related symptoms? Is treatment compliance similarly higher among these individuals?

**Response: The objective of this paper was mainly to document IVM coverage and changes over time. Unfortunately, our methods in the current study did not permit us to capture the clinical data requested by the reviewer. Other papers by our group have detailed the clinical features of onchocerciasis/epilepsy in the Western Equatoria State of South Sudan and how they relate with ivermectin coverage. See for example the following publications:**

- Raimon S, Dusabimana A, Abd-Elfarag G, Okaro S, Carter JY, Newton CR, et al. High Prevalence of Epilepsy in an Onchocerciasis-Endemic Area in Mvolo County, South Sudan: A Door-To-Door Survey. Pathogens. 2021;10: 599. doi:10.3390/pathogens10050599
- Jada SR, Dusabimana A, Abd-Elfarag G, Okaro S, Brusselaers N, Carter JY, et al. The Prevalence of Onchocerciasis-Associated Epilepsy in Mundri West and East Counties, South Sudan: A Door-to-Door Survey. Pathog Basel Switz. 2022;11: 396. doi:10.3390/pathogens11040396
- Colebunders R, Abd-Elfarag G, Carter JY, Olore PC, Puok K, Menon S, Siewe Fodjo JN, et al. Clinical characteristics of onchocerciasis-associated epilepsy in villages in Maridi County, Republic of South Sudan. Seizure 2018; 62: 108–115. doi: 10.1016/j.seizure.2018.10.004.

**We suppose that persons with onchocerciasis skin lesions and itching may also be more likely to take ivermectin to attenuate the symptoms. However, we did not investigate this in Maridi. Questions about itching, blindness and ivermectin use were asked in a survey in two other onchocerciasis endemic areas in South Sudan. These data will be presented in a additional paper concerning ivermectin coverage in South Sudan.**
